# Predicting responsiveness to a sustained reading and spelling intervention in children with dyslexia

**DOI:** 10.1002/dys.1614

**Published:** 2019-04-23

**Authors:** Elisabeth A.T. Tilanus, Eliane Segers, Ludo Verhoeven

**Affiliations:** ^1^ Behavioural Science Institute Radboud University Nijmegen The Netherlands; ^2^ Marant, Elst Gelderland The Netherlands

**Keywords:** dyslexia, initial abilities, initial treatment success, precursor measures, primary school, responsiveness to intervention

## Abstract

The present study aimed to predict responsiveness to a sustained two‐phase reading and spelling intervention with a focus on declarative and procedural learning respectively in 122 second‐grade Dutch children with dyslexia. We related their responsiveness to intervention to precursor measures (phonological awareness, rapid automatized naming ability, letter knowledge, and verbal working memory) and related word and pseudoword reading and spelling outcomes of the sustained intervention to initial reading and spelling abilities, and first‐phase, initial treatment success. Results showed that children with dyslexia improved in reading accuracy and efficiency and in spelling skills during the two phases of the intervention although the gap with typical readers increased. In reading efficiency, rapid automatized naming, and in reading and spelling accuracy phoneme deletion predicted children's responsiveness to intervention. Additionally, children's initial reading abilities at the start of the intervention directly (and indirectly, via initial treatment success, in reading efficiency) predicted posttest outcomes. Responsiveness to intervention in spelling was predicted by phoneme deletion, and spelling at posttest was indirectly, via initial treatment success, predicted by children's initial spelling abilities. Finally, children's initial treatment success directly predicted reading efficiency and spelling outcomes at posttest.

## 1 INTRODUCTION

Dyslexia is a reading disorder that, by definition, is difficult to remediate (American Psychiatric Association, 2013). Children with dyslexia have both reading accuracy and reading fluency problems, and often also spelling problems (Bates et al., 2007; Ehri, 2000). Across different orthographies, reading and spelling interventions for poor readers generally focused on gaining declarative knowledge to become fully accurate in word decoding. Only very few interventions included procedural aspects to improve fluency (Struiksma, van der Leij, & Stoel, 2009; Zoccolotti, De Luca, Marinelli, & Spinelli, 2014). This is particularly remarkable in the case of transparent orthographies, as children with dyslexia in these orthographies are not so much inaccurate but rather slow (de Jong & van der Leij, 2003; Furnes & Samuelsson, 2011; Torgesen, Rashotte, & Alexander, 2001; Wimmer, 1993; Zoccolotti et al., 2014). Therefore, sustained interventions would ideally include not only declarative but also procedural aspects of reading. Severe problems in reading and spelling are often related to individual differences in phonological awareness, rapid automatized naming, letter knowledge, and verbal working memory, but to what extent such individual differences account for variation in responsiveness to intervention is far from clear. Moreover, there is little insight in the role of children's initial reading and spelling abilities and in initial treatment success in the explanation why children respond differently to interventions. In order to shed more light on the responsiveness to a sustained intervention, the present study examined to what extent the outcomes of a two‐phase reading and spelling intervention focusing on declarative and procedural learning respectively could be predicted by (a) precursor measures, (b) initial reading and spelling abilities at the start of the intervention, and (c) initial treatment success after the declarative phase of the intervention.

Most existing reading interventions that are described in the literature are short term and classroom based. The focus of clinical interventions is mostly on the improvement of accurate decoding skills (Lovett, Barron, & Benson, 2003). A frequently used explicit and declarative approach in the remediation of children with dyslexia is a phonics‐based instruction with a strong focus on phonological recoding. This has proved to be effective to improve children's decoding skills (Chen & Savage, 2014; Devonshire, Morris, & Fluck, 2013; Fuchs & Vaughn, 2012; Galuschka, Ise, Krick, & Schulte‐Körne, 2014; Moats, 2009; Snowling & Hulme, 2011; Tilanus, Segers, & Verhoeven, 2016; Watson & Jonston, 2005). To improve word reading fluency, generally defined as the ability to read single words fast and relatively effortless (Torgesen, Rashotte, Alexander, Alexander, & McPhee, 2003), repeated word reading, in which a set of words is read out aloud repeatedly is often used (Kuhn & Stahl, 2003). Many fluency‐oriented reading experiments have succeeded in improving word reading speed of trained items. However, the challenge of fluency‐oriented interventions is to transfer the outcomes to untrained items (Berends & Reitsma, 1998; Van Gorp, Segers, & Verhoeven, 2017). Based on the assumption that reading fluency is dependent on accurate word decoding (Barth, Catts, & Anthony, 2009; Torgesen et al., 2001; Zoccolotti et al., 2014), interventions would ideally be sustained, including declarative and procedural phases.

In addition to learning to read, learning to spell is one of the major achievements at primary school (Moats, 2009). There is great overlap in precursor measures related to reading and spelling in children with dyslexia (Apel, 2009; Coltheart, Rastle, Perry, Langdon, & Ziegler, 2001; Ehri, 2000). Given the fact that reading and spelling share common underlying knowledge sources, it is assumed that learning grapheme–phoneme relationships will strengthen phoneme–grapheme relationships and vice versa (Van Orden, 1987; Ehri, 2014). However, spelling is less well studied than reading and reading, and spelling interventions are frequently described in separate studies. Although it can be argued that spelling is more difficult than reading (Bosman & van Orden, 2003), learning to read and learning to spell are closely related and should ideally be taught together and interventions focusing on both reading and spelling can thus be recommended (see Ehri, 2000).

The main goal of predicting responsiveness to intervention is to become aware how individual differences account for differential intervention outcomes. Individual variation in phonological awareness, rapid automatized naming, letter knowledge, and verbal working memory have often found to be related to severe reading and spelling problems (Frijters et al., 2011; Scheltinga, van der Leij, & Struiksma, 2010; Tijms, 2011; Tilanus, Segers, & Verhoeven, 2013). There is strong evidence that phonological awareness and naming speed, as assessed by rapid automatized naming tasks, are related to severe problems in reading fluency (Fletcher et al., 2011; Frijters et al., 2011; Kuhn & Stahl, 2003; Zoccolotti et al., 2014). Whereas children without dyslexia become accurate and fluent readers after repeated reading of words in text, children with dyslexia show persistent problems with the automatization and still read letter by letter. A prerequisite, before one can speed up, is having full letter knowledge. Many children with poor reading skills have problems with learning the grapheme–phoneme correspondences that are required to decode words (Barth et al., 2009; Ehri, 1997; Ehri, 2005; Gottardo, Chiappe, Siegel, & Stanovich, 1999). Children's verbal working memory has also found to be related to persistent reading and spelling problems (Berninger, Abbott, & Vermeulen, 2002; Frijters et al., 2011; Fuchs et al., 2012; Lovett et al., 2003). Several studies have confirmed the role of working memory in children's reading skills (Jongejan, Verhoeven, & Siegel, 2007; Russell, 2002). It is generally assumed that working memory plays a direct or indirect role in the establishment of reading fluency (Barth et al., 2009). Both phonological awareness (Fletcher et al., 2011; Frijters et al., 2011; Otaiba & Fuchs, 2002) and naming speed have also been evidenced as predictors of responsiveness to intervention (Barth et al., 2009; Jongejan et al., 2007; Misra, Katzir, & Wolf, 2004). Tijms (2011), for example, found that rapid automatized naming and phonological memory showed a relatively consistent impact on the effectiveness of a Dutch dyslexia treatment. Scheltinga et al. (2010) demonstrated that pretest reading accounted for a modest part of the variance at posttest. Thus, the understanding of the role of precursor measures is important not only in identifying reading and spelling problems but also in relation to children's responsiveness to intervention (Beach & Rollanda, 2015; Denton et al., 2013; Elliott & Grigorenko, 2014; Fletcher et al., 2011; Otaiba & Fuchs, 2002; Wanzek & Vaughn, 2009). It would help to understand why some children are more resistant to reading and spelling interventions than others (Arfe, Dockrell, & Berninger, 2014; Furnes & Samuelsson, 2011).

In order to shed more light on the individual variation in responsiveness to sustained intervention, we examined a two‐phase reading and spelling intervention, consisting of an initial, declarative, and a follow‐up procedural phase. We investigated the responsiveness to intervention in 122 second‐grade children with dyslexia in the Netherlands. Both after the first and second phase of the intervention, children's responsiveness was measured on their accuracy and efficiency in word and pseudoword reading and their accuracy of word spelling. Children's responsiveness was related to their phonological awareness, rapid automatized naming abilities, letter knowledge, and verbal working memory. Moreover, children's initial reading and spelling abilities at the start of the intervention and their treatment success after Phase 1 of the intervention were added to the prediction of children's reading and spelling outcomes by the end of the sustained intervention. Accordingly, two research questions were addressed in the present study.

1. To what extent do children with dyslexia profit from a two‐phase sustained reading and spelling intervention?
2. How do phonological awareness, rapid automatized naming, letter knowledge, and verbal working memory predict children's responsiveness to the sustained intervention, and how are initial reading and spelling abilities at the start of the intervention and initial treatment success related to reading and spelling outcomes at posttest?

To find an answer to the first question, we compared the dyslexic children's reading and spelling skills at the start and after the first and second phases of the intervention with those of 108 typical reading peers. We expected catching‐up effects for accuracy (both in word and pseudoword decoding and in word spelling) but less so for efficiency. In order to answer the second question, we used a mediation analysis (Hayes, 2013) to investigate to what extent (a) phonological awareness, rapid automatized naming, letter knowledge and verbal working memory, (b) initial reading and spelling abilities, and (c) initial treatment success are related to children's reading and spelling outcomes at posttest. We expected that children's reading and spelling accuracy could be predicted by phonological awareness, and their reading efficiency by rapid automatized naming. Furthermore, we expected that children's initial reading and spelling abilities as well as their initial treatment success would be additional predictors of their reading and spelling outcomes of the sustained intervention.

## 2 METHOD

### 2.1 Participants

Two groups of children participated in this study and were assessed at three times. The first evaluation was at the time of diagnostics (Time 1), followed by an evaluation after the first phase of the intervention, consisting of 12 treatment sessions (Time 2; see Tilanus et al., 2016). The final evaluation took place after the second phase of the intervention, which consisted of 36 additional treatment sessions (i.e., after a total of 48 treatment sessions; Time 3). The first group were Dutch second graders with diagnosed developmental dyslexia (*N* = 122). They were referred to a clinic by their parents following advice from the school. This group started intervention throughout second grade. Children's waiting time, the number of days between initial assessment and treatment, varied and was therefore taken into account (*M* = 80.44 days, *SD* = 34.03 days). The group consisted of 75 boys and 47 girls with an age ranged from 7 to 8 years at pretest and 83 children repeated a class. All children met the formal criteria of dyslexia in accordance with the definition of the Dutch Dyslexia Foundation (Stichting Dyslexie Nederland, 2008) and the American Psychiatric Association (2013). According to this definition, dyslexia is an impairment characterized by persistent problems in learning to read and/or write words or in the automatization of reading and writing. The level of reading and/or writing has to be significantly lower than what can be expected based on the educational level and age of the individual.

The control group consisted of 108 typical readers (56 boys, 52 girls) in second grade with an age between 6 to 8 years old at pretest. Three children had repeated a class. This group was selected randomly out of the lists of schools of the children with dyslexia to function as a control group.

### 2.2 Measures

#### 2.2.1 Decoding

Children's decoding skills were measured with two tests. Reading real words was assessed by the *Brus Eén Minuut Test* [Brus 1‐min test] (Brus & Voeten, 1973). In this time‐limited test (1 min), the efficiency and accuracy of reading unrelated words, increasing in length, were measured. Reading pseudowords was assessed by the *Klepel* (Van den Bos, Lutje Spelberg, Scheepstra, & de Vries, 1994). In this test, children have to read as many unrelated pseudowords as possible in 2 min. In both tests, the efficiency score was calculated by the total number of read words minus the number of errors and the accuracy score was calculated by the percentage of (pseudo)words read correctly. Both tests were conducted three times. Both reading tests consisted of two parallel tests, Form A and Form B. Children received Form A at Time 1 and Time 3. Form B was used at Time 2.

#### 2.2.2 Spelling

Spelling skills were assessed using the *PI‐woorddictee* [word dictation] (Geelhoed & Reitsma, 1999). This test contains a word dictation for the investigation of the spelling skills in writing single words. Two parallel forms were used, Form A (at Time 1 and Time 3) and Form B (at Time 2). The test consisted of nine blocks of 15 words (the maximum score is 135). The words were read aloud in a sentence, and the child was asked to write down the repeated word. No verbs are used. When a child had at least eight words written incorrectly, the test was discontinued. Scoring is based upon the number of written words correctly.

#### 2.2.3 Phonological awareness

Children's phonological awareness was measured by two subtests of *DST* ^*NL*^: *Letterverwisseling* [phoneme manipulation] and *Klanksplitsing* [phoneme deletion] (Kort et al., 2005). In phoneme manipulation, the child was asked to change the switch the initial sounds of two words. The raw score consisted of the number of correctly read words (maximum score was 11). In phoneme deletion, the child was asked to speak out the word that remained when a particular phoneme was deleted. Maximum score on this task was 12. These tests could only be interpreted in terms of accuracy. To make these tests comparable, we used weighted scores.

#### 2.2.4 Rapid automatized naming

Children's naming speed was measured by the subtest *Cijfers Benoemen* [naming digits] of *Continu Benoemen & Woorden Lezen* (*CB&WL*) [continuous naming and reading words] (van den Bos & Lutje Spelberg, 2007). The child has to read as fast as possible out loud 50 digits (five rows of 10 digits). The time (in seconds), which was needed to read all digits, was used for analysis. Therefore, a high score indicates a weak performance on the task.

#### 2.2.5 Letter knowledge

Letters benoemen [letter naming] (Struiksma, van der Leij, & Vieijra, 1997) was used to examine children's grapheme–phoneme association. Children have to read out loud as accurate and as fast as possible 36 graphemes, which were shown on a card. The number read correctly was used in the present study.

#### 2.2.6 Verbal working memory

The Dutch version of the Digit Span subtest *Cijferreeksen* [digit span] (VWM) from *WISC‐III* ^*NL*^ (Kort et al., 2005) was used to measure children's verbal working memory. The number of digits that the child is able to repeat in correct or reversed serial order immediately after hearing them represents the score on this test. The test consists of two parts. The first eight items, with an increasing difficulty must be repeated in the correct order. For each item, a child had two attempts. Children received one point for each well repeated digit span. The maximum score on this part of the test is 16. Failed on both attempts (two times score zero), the first part of the test was discontinued. The test was continued by digit span in the reverse order. For naming digits backwards, seven items were available, with again two attempts for each item. The same cut‐off point was used as in naming digits in the same order (maximum score 14). The raw score, number of correct answers (minimum 0, maximum 30) was converted into a standardized score, which is used for analysis.

### 2.3 Procedure and intervention

Children were referred to the clinic when reading and spelling arrears were observed at school. These arrears existed despite of additional and intensive remediation at school. Each child was tested individually at the clinic by a certified MSc‐graduated clinician. Pretesting (Time 1) was done in the beginning of Grade 2. Children were eligible for the intervention when they met the criteria according the *Protocol Dyslexie Diagnostiek en Behandeling* [Protocol Dyslexia Diagnostic and Treatment] (Blomert, 2006). For children who met these criteria, a two‐phase reading and spelling intervention was set up. The main goal of the intervention was to achieve a functional level of technical reading and spelling. The two‐phase intervention assumed a specific language processing problem in children with dyslexia, often characterized with a phonological deficit. Accordingly, the first phase of the intervention is a declarative approach, which focused explicitly on learning the grapheme–phoneme and phoneme–grapheme correspondences using a phonics‐based intervention during the first 12 treatment sessions (see Tilanus et al., 2016). For those children who had very poor grapheme–phoneme knowledge, a nonalphabetical approach would have been used in which the focus is on the sound structure of words explicitly. This was not the case in the current sample.

In the first phase of the intervention, only grapheme‐phoneme correspondence (GPCs) and monosyllabic words were used. The focus was on the phonetic structure of Dutch words. The intervention starts with the focus as in regular phonics intervention approaches. Children learn to distinguish phonemes into categories (e.g., short vowels, long vowels, digraph, trigraphs, quadrugraphs, and consonants). The categories are represented in a sound schedule (see Tilanus et al., 2016). The schedule contained pure sounds, no combined sounds or letter clusters as for example “sch” (which is a cluster of the phonemes/s/, a consonant and/ch/, a digraph) and “‐ing” (which is a cluster of the phonemes/i/, a short vowel and/ng/, a digraph). Although these letter clusters are not included in the sound schedule, they were addressed during the intervention when children learn to combine categories. Additionally to the regular approaches, symbolic scaffolds were added to simplify the structure of GPCs. Mnemonic cards were used to optimize these links.

During each treatment session and in the related home exercises, children practiced to connect and recognize phoneme strings, letter clusters, and morphemes. The symbolic scaffolds, which they have learned at the start of the intervention help them to identify the structure of words more easily and faster. The first phase of the intervention thus focused on the GPC‐level to optimize the foundation level of reading and on the sublexical level to strengthen the ability to combine phonemes and recognize clusters of words and morphemes in monosyllabic and—in the next stage of the intervention—polysyllabic words. After 12 treatment sessions, children received a repeated measure to evaluate their reading and spelling skills (Time 2). This initial treatment success is defined as children's change in skills in the period from Time 1 to Time 2.

**Figure 1:**
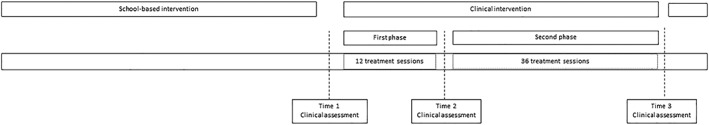
Timeline with the different phases and duration of clinical assessment and intervention

The second phase of the intervention is a sustained approach during a prolonged time and focuses on declarative and procedural aspects and the reading and spelling of disyllabic and polysyllabic words. Procedural aspects were added to the initial declarative approach. During 36 sessions, the focus shifts from specific GPC learning and monosyllabic word reading and spelling to applying this knowledge in polysyllabic words. Besides accuracy, reading efficiency plays a more important role in the intervention programme, using word flashcards. As dysfluency is the main characteristic of children with dyslexia in transparent orthographies such as Dutch (Struiksma et al., 2009), the second phase of the intervention consisted of an additional procedural approach. In contrast to many school‐based interventions, the approach uses syllabic segmentation, starting out from phonological units, instead of syllables to read and spell polysyllabic words adequately and easily (e.g., the word “butter” is divided in two phonological units: /bu/ and/tter/instead of/but/and/ter/as when using syllables). So‐called sound bars represent the number of phonological units in a word. At each sound bar, symbolic scaffolds are placed to help children to recognize clusters in words relatively effortlessly. At the same time, symbolic scaffolds will help children to understand why, for example, vowels are pronounced as a long or short vowel and should be written with a specific spelling rule. The second phase of the intervention builds on the first phase of the intervention. The sustained intervention is an explicit declarative approach at the first phase and includes an additional procedural approach in the second phase. Posttest (Time 3) took place after children passed through the entire intervention. The second phase of the intervention is defined as the period from Time 2 to Time 3. The different phases and the duration of each phase are represented in a timeline in Figure 1.

The individual treatment sessions took 45 min weekly with a clinician. All clinicians completed a training at the clinic in order to deliver the intervention programme. The continuity of the quality of the intervention was guaranteed by four training sessions per year. Treatment sessions consisted of five parts (retrieving knowledge of previous sessions, introducing a new GPC or spelling rule, reading and spelling by guided exercise, and improving reading efficiency by using flashcards and text reading). Besides these weekly sessions with the clinician, children were required to practice at home four times a week for 20 min for reading and two times a week for 10 min for spelling. Parents and teachers were informed by special parent‐written manuals and explanations for each exercise. Parents and teachers informed the clinician weekly by describing the progress of the home exercises in online logbooks. If the home exercises were not done, the intervention could be ended directly in order to be ensured of the continuity and fidelity of the intervention. This was not the case in the present study.

All data were documented in an online database and were evaluated by a team of responsible certified psychologists at the clinic.

## 3 RESULTS

As a preliminary check, we tested whether children with dyslexia differ from typical readers at Time 1, Time 2, and Time 3 and whether children with dyslexia improve their reading and spelling skills over time. Children with dyslexia were behind on all measures each time compared with their typical reading peers (see Table 1).

**Table 1:** Descriptive statistics for typical readers and children with dyslexia at Time 1, Time 2, and Time 3

		Typical readers		Children with dyslexia			
		N	M (SD)	N	M (SD)	t	d
(Time 1) Precursor measures							
Letter knowledge	Letter naming	108	34.77 (1.66)	122	33.40 (2.27)	−5.25***	0.69
Rapid automatized naming	Digits	108	35.31 (6.83)	119	42.88 (11.66)	6.03***	0.87
Verbal working memory	Repeating digits	107	10.30 (2.71)	121	7.91 (2.52)	−6.89***	−0.92
Phonological awareness	Deletion	108	6.44 (2.30)	120	5.77 (2.22)	−2.22*	−0.30
	Spoonerism	108	3.03 (3.18)	119	0.79 (1.62)	−6.57***	−1.05
Decoding efficiency							
Time 1	Words	108	75.46 (30.17)	122	32.02 (13.95)	−13.72***	−2.27
	Pseudowords	108	30.49 (15.08)	122	11.90 (5.38)	−12.14***	−2.12
Time 2	Words	105	88.80 (26.83)	120	46.30 (21.31)	−13.03***	1.75
	Pseudowords	105	33.49 (13.33)	120	16.47 (8.61)	−11.20***	1.52
Time 3	Words	95	118.69 (27.61)	86	66.42 (23.50)	−13.76***	2.04
	Pseudowords	95	55.38 (18.65)	95	23.51 (10.18)	−14.45***	2.12
Decoding accuracy							
Time 1	Words	108	90.61 (9.84)	115	78.64 (13.84)	−7.48***	−1.04
	Pseudowords	108	61.08 (18.60)	118	48.57 (17.67)	−5.19***	−0.70
Time 2	Words	105	93.52 (6.67)	119	84.29 (11.01)	−7.68***	1.02
	Pseudowords	105	63.09 (16.15)	119	56.03 (16.66)	−3.21**	0.43
Time 3	Words	95	96.93 (3.36)	86	90.03 (7.62)	−7.75***	1,17
	Pseudowords	95	84.28 (12.14)	86	61.85 (16.47)	−10.34***	1.55
Spelling							
Time 1	Word dictation	108	36.44 (16.83)	122	23.48 (12.18)	−6.62***	−0.95
Time 2	Word dictation	105	52.09 (17.60)	119	41.08 (11.72)	−5.43***	0.74
Time 3	Word dictation	95	79.72 (23.11)	85	63.31 (17.24)	−5.44***	0.81

* *p* < .05.

** *p* < .01.

*** *p* < .001.

Within the group of children with dyslexia, positive changes in skills were found. Children with dyslexia showed a significant change in skills between Time 1 and Time 2, between Time 2 and Time 3, and between Time 1 and Time 3 (see Table 2).

**Table 2:** Children's changes in skills between Time 1–2, Time 2–3, and Time 1–3 on word decoding efficiency (WDE), word decoding accuracy (WDA), pseudoword decoding efficiency (PWDE), pseudoword decoding accuracy (PWDA), and spelling

	Time 1–2			Time 2–3			Time 1–3		
	t	df	d	t	df	d	t	df	d
WDE	−10.87^***^	119	−1.99	−11.37^***^	85	−2.47	−20.05^***^	85	−4.35
WDA	−4.49^***^	111	−0.85	−4.25^***^	85	−0.92	−8.73^***^	82	−1.93
PWDE	−7.64^***^	119	−1.4	−7.72^***^	85	−1.67	−13.46^***^	85	−2.92
PWDA	−4.73^***^	114	−0.89	−2.60^*^	85	−0.56	−6.38^***^	83	−1.4
Spelling	−18.45^***^	118	−3.4	−15.34^***^	84	−3.35	−24.50^***^	84	−5.25

### 3.1 Change in reading and spelling skills

We investigated children's change in skills by conducting several general linear model repeated measure analyses, taking the multivariate approach, and exploring differences between typical readers and children with dyslexia over Time on decoding (efficiency and accuracy, words, and pseudowords) and word spelling. Group (typical readers vs. children with dyslexia) was the between subjects factor each time. Time (Time1, Time 2, and Time 3) and Word (words vs. pseudowords) were the within subjects factors.

### 3.2 Decoding efficiency

For efficiency, we found main effects of Time and Group as well as an interaction between Time*Group. Typical readers outperformed children with dyslexia at Time1, Time 2, and Time 3. We also found a main effect of Word, an interaction between Time*Word, an interaction between Word*Group, and an interaction between Time*Word*Group. We conducted separate analyses for word and pseudoword decoding efficiency to further disentangle the Time*Word*Group (see Table 3).

**Table 3:** Results of the general linear model repeated measure analyses on decoding (efficiency, accuracy, words, and pseudowords) and spelling

		F (df)	p	η ^2^_p_
Decoding efficiency	Time	505.121 (2,178)	<.001	.85
	Time*Group	19.728 (2,178)	<.001	.18
	Word	1,909.502 (1,179)	<.001	.91
	Word*Group	159.290 (1,179)	<.001	.47
	Time*Word	259.379 (2,178)	<.001	.75
	Time*Word*Group	8.730 (2,178)	<.001	.09
Words	Time	1,049.169 (1,179)	<.001	.85
	Group	207.432 (1,179)	<.001	.54
	Time*Group	5.869 (1,179)	.016	.03
Pseudowords	Time	579.165 (1,179)	<.001	.76
	Group	182.847 (1,179)	<.001	.51
	Time*Group	55.421 (1,179)	<.001	.24
Decoding accuracy	Time	118.638 (2,175)	<.001	.43
	Time*Group	8.166 (2,175)	<.001	.05
	Word	1,346.898 (1,176)	<.001	.88
	Word*Group	11.165 (1,176)	.001	.06
	Time*Word	36.553 (2,175)	<.001	.17
	Time*Word*Group	32.633 (2,175)	<.001	.15
Words	Time	129.704 (1,176)	<.001	.42
	Group	103.471 (1,176)	<.001	.37
	Time*Group	17.373 (1,176)	<.001	.09
Pseudowords	Time	190.973 (1,177)	<.001	.52
	Group	56.860 (1,177)	<.001	.24
	Time*Group	16.941 (1,177)	<.001	.09
Word spelling accuracy				
Spelling	Time	1,287.630 (1,178)	<.001	.88
	Group	43.835 (1,178)	<.001	.20
	Time*Group	0.819 (1,178)	.367	.01

For word decoding efficiency, the differences in growth between Time 1 and Time 2 did not differ between typical readers and children with dyslexia. However, the difference in growth between Time 2 and Time 3 was significant *t*(163.417) = −3.187, *p* = .002, *d* = −0.50, in advantage for typical readers. Overall, the groups differ in growth between Time 1 and Time 3 in advantage for typical readers *t*(179) = −2.423, *p* = .016, *d* = −0.36. Within the group of children with dyslexia, the difference growth between Time 1 and Time 2 versus Time 2 and Time 3 was significant *t*(85) = −2.273, *p* = .026, *d* = −0.49. Children with dyslexia showed more progression during the final period.

For pseudoword decoding efficiency, the difference in growth was significant, in advantage for children with dyslexia during Time 1 to Time 2 *t*(223) = 2.016, *p* = .045, *d* = 0.27. Between Time 2 and Time 3, typical readers showed more progression than children with dyslexia *t*(179) = −9.24, *p* < .001, *d* = −1.38. Overall, the groups differ in the growth between Time 1 and Time 3 in advantage for typical readers *t*(169.320) = −8.418, *p* = <.001, *d* = −1.29. The differences in growth within the group of children with dyslexia did not differ between Time 1 and Time 2 versus Time 2 and Time 3 *t*(85) = −1.803, *p* = .075, *d* = −0.39.

Finally, we checked whether the intervention showed normalization effects. Therefore, we converted the scores at each time point for the typical readers and children with dyslexia into *z*‐scores and compared the *z*‐scores of the children with dyslexia at Time 1 versus Time 3. For word decoding efficiency, there was no difference in *z*‐scores between Time 1 and Time 3 *t*(85) = 1.84, *p* = .069, *d =* 0.40, and for pseudoword decoding efficiency, there was a decline *t*(85) = 3.80, *p* = .001, *d* = 0.82. This declining *z*‐score indicated no normalization effect at this point. However, it should be noted that the percentage of children who belonged to the lowest 10% scoring children decreased for both word decoding efficiency (−18%) and pseudoword decoding efficiency (−9%).

### 3.3 Decoding accuracy

For accuracy, we found main effects of Time and Group as well as an interaction between Time*Group. Children with dyslexia were behind typical readers at Time 1, Time 2, and Time 3. We also found a main effect of Word, an interaction between Time*Word, an interaction between Word*Group, and an interaction between Time*Word*Group. We conducted separate analyses for word and pseudoword decoding accuracy to further disentangle the Time*Word*Group interaction (see Table 3).

For word decoding accuracy, the difference in growth was only significant between Time 1 and Time 2 *t*(215) = 2.05, *p* = .04, *d* = 0.28. Children with dyslexia showed more progression during this time than the control group. Overall, the groups differ in the growth between Time 1 and Time 3 in advantage for children with dyslexia *t*(133.414) = 4.045, *p* = <.001, *d* = 0.70. For children with dyslexia, their growth between Time 1 and Time 2 versus Time 2 and Time 3 did not differ *t*(82) = 0.674, *p* = .502, *d* = 0.15.

For pseudoword decoding accuracy, the difference in growth was significant too between Time 1 and Time 2 *t*(212.21) = 2.68, *p* = .008, *d* = 0.37, and between Time 2 and Time 3 *t*(179) = −5.64, *p =* <.001, *d* = −0.84, in advantage for typical readers. Overall, the groups differ in the growth between Time 1 and Time 3 in advantage for typical readers *t*(177) = −4.116, *p* < .001, *d* = −0.62. Within the group of children with dyslexia, the growth between Time 1 and Time 2 versus Time 2 and Time 3 did not differ *t*(83) = 0.669, *p* = .505, *d* = 0.15.

Also here, the scores of the children at Time 1 and Time 3 were transformed into *z*‐scores. In the group of children with dyslexia, no significant difference was found for word decoding accuracy *t*(82) = 0.93, *p* = .353, *d* = 0.21, and again a decline for pseudoword decoding accuracy *t*(83) = 4.07, *p* = .001, *d* = 0.89. No normalization effect was thus found. However, the number of children who belonged to the lowest 10% on word decoding accuracy decreased (−6%). For pseudoword decoding accuracy a small, but negligible, increase was found (+1%).

### 3.4 Spelling

Finally, for word spelling, we found a main effect of Time and Group. We did not find an interaction between Time and Group. Children with dyslexia were behind typical readers all times. Overall, the groups did not differ in the growth between Time 1 and Time 3 *t*(178) = −0.905, *p* = <.367, *d* = −0.14. However, the differences in growth between Time 1 and Time 2 versus Time 2 and Time 3 within the group of children with dyslexia were significant *t*(84) = −2.863, *p* = .005, *d* = −0.63. Children with dyslexia showed more growth between Time 2 and Time 3 compared with their growth between Time 1 and Time 2.

Regarding spelling, no significant difference was found when comparing *z*‐scores between Time 1 and Time 3 *t*(84) = −0.09, *p* = .926, *d* = −0.02. However, the number of children who belonged to the lowest 10% decreased (−3%).

### 3.5 Individual differences in response to intervention

With respect to the second research question, we investigated to what extent responsiveness to intervention within the group of children with dyslexia is related to precursor measures and to what extent reading and spelling outcomes at posttest can be explained by children's initial reading and spelling abilities (Time 1) and children's initial treatment success (Time 2). We used the Process add‐on in SPSS (Hayes, 2013) and performed a mediation analysis. Scores at Time 1 and the precursors letter knowledge (LK), rapid automatized naming, verbal working memory (VWM), phoneme manipulation (PM), and phoneme deletion (PD) were the independent variables. Time 2 scores on word and pseudoword decoding (efficiency and accuracy) and word spelling were the mediators, and the Time 3 score on these measures was the dependent variable. The model was run seven times, each with six independent variables as covariate, to be able to estimate the effects. Waiting time (WT) was added as covariate for each analysis. Bootstrapping was set at 5,000 cycles, as recommended by Hayes (2013).

**Figure 2:**
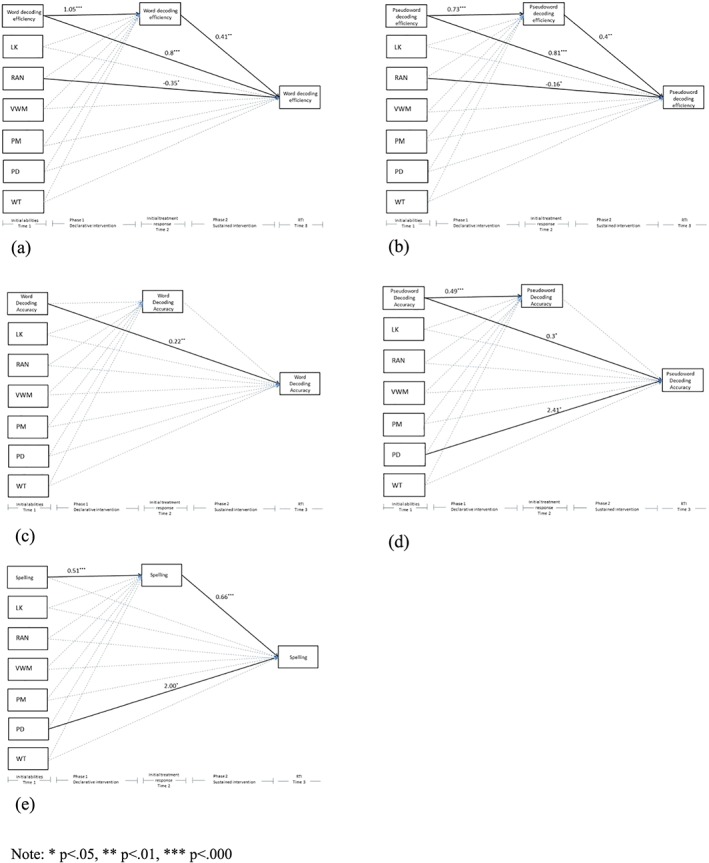
Model for predicting posttest scores on (a) word decoding efficiency, (b) pseudoword decoding efficiency, (c) word decoding accuracy, (d) pseudoword decoding accuracy, and (e) spelling. Significant coefficients are reported [Colour figure can be viewed at wileyonlinelibrary.com]

Figure 2 depicts five models. The *R* ^2^ of all models was significant. The first two models, model (a) *R* ^2^ = 0.66, *p* < .001, and model (b) *R* ^2^ = 0.56, *p* < .001, are related to word and pseudoword decoding efficiency and model (c) *R* ^2^ = 0.33, *p* < .001, and model (d) *R* ^2^ = 0.34, *p* < .001, are related to word and pseudoword decoding accuracy. The final model (e) *R* ^2^ = 0.47, *p* < .001, is related to the accuracy of word spelling.

### 3.6 Decoding efficiency

For both efficiency measures, direct and indirect effects were found. For word decoding efficiency, Time 1 word decoding efficiency showed an indirect effect via Time 2 [0.1002; 0.9668] (Figure 2a), and for pseudoword decoding efficiency, Time 1 pseudoword decoding efficiency showed an indirect effect via Time 2 [0.0770; 0.6773] (Figure 2b).

In addition to children's initial word and pseudoword efficiency level, the precursor measure rapid automatized naming and children's initial treatment success directly predicted Time 3 outcomes.

### 3.7 Decoding accuracy

For word decoding accuracy and pseudoword decoding accuracy, Time 1 was a direct predictor of Time 3, no indirect effects were found (see Figures 2c,d). For pseudoword decoding accuracy also the precursor, phoneme deletion showed a direct effect (Figure 2d).

### 3.8 Spelling

An indirect effect of Time 1 spelling via Time 2 was found for Time 3 [0.1272; 0.6809]. Children's initial spelling level did not directly predict Time 3 outcomes. A direct effect of the precursor measure phoneme deletion on Time 3 spelling was found.

## 4 DISCUSSION AND CONCLUSION

The main aim of the present study was to predict responsiveness to a sustained two‐phase reading and spelling intervention in children with dyslexia. The intervention consisted of an initial and declarative stage focusing on reading and spelling accuracy, and a follow‐up declarative and procedural stage focusing on reading and spelling fluency. We investigated children's initial reading and spelling abilities at the start of the intervention and examined their posttest outcomes to the first and second phase of the intervention in terms of accuracy and efficiency in word decoding, pseudoword decoding, and in word spelling accuracy. We compared the scores of children with dyslexia with the scores of a typical reading group. We addressed two research questions.

Our first research question was to what extent children with dyslexia profited from a two‐phase sustained reading and spelling intervention. As expected, children with dyslexia improved their reading and spelling skills during the two phases of the intervention. In particular, they improved their word and pseudoword decoding accuracy and their pseudoword decoding efficiency during the initial declarative approach. These findings imply positive changes in skills in children with dyslexia during the first phase of the intervention and is in line with other studies which evidenced that a declarative approach is helpful for children with dyslexia to improve their reading and spelling abilities (Devonshire et al., 2013; Galuschka et al., 2014; Tilanus et al., 2016). Moreover, during the second, declarative and procedural phase in the sustained intervention children with dyslexia further improved their reading and spelling abilities. Significant changes were found between children's abilities at the beginning and after the second phase of the intervention for word and pseudoword decoding accuracy and efficiency as well as for word spelling accuracy.

When we compared the scores of children with dyslexia with the scores of a typical reading it was evidenced that the group of typical readers outperformed children with dyslexia at the start of the intervention, after the initial declarative approach and after the declarative and procedural phase in the sustained intervention on all measures. The fact that typical readers improved their reading skills over time, in such a way that they outperformed children with dyslexia not only at Time 1, but also at Time 2 and Time 3, contributes to the explanation why we did not find strong normalization effects, albeit that less children were in the groups of the lowest 10% scorers. The growth between the two groups during the two phases of the intervention differs. Children with dyslexia improved their reading and spelling skills especially during the first phase of the intervention. In this initial stage, their change in reading and spelling skills was comparable with typical readers or even better. The fact that children received a number of 12 treatment sessions in the first phase of the intervention, while they received a number of 36 treatment sessions in the second phase, could probably contribute to the explanation why typical readers outperform children with dyslexia during the second phase of the intervention. Although children with dyslexia showed a relatively small growth during the second part of the intervention, their typical reading peers still improved their skills. For reading efficiency, this is consistent with results of other studies in which the lack of fluency is characterized as the most persistent and significant characteristic of developmental dyslexia (de Jong & van der Leij, 2003; Kuhn & Stahl, 2003; Struiksma et al., 2009; Wimmer, 1993; Žarić et al., 2014). With respect to spelling, this is in contrast with the results by Tijms (2011), who found normalization on spelling ability after a comparable intervention approach albeit in an older age group. The age differences in participants between the two studies could explain the effect as older children can better relate the intervention to what is being taught at school. Although children with dyslexia in the present study did not show normalization effects, the absolute number of children who escaped the 10% cut‐off criterion, which is one of the criteria to diagnose a child as having dyslexia (see Blomert, 2006), increased on all measures, except pseudoword decoding accuracy. The number of children with severe reading and spelling problems thus decreased in absolute numbers. Overall, these results are in line with existing literature that children with dyslexia show severe and persistent problems with reading and spelling despite remediation. However, the finding that the number of children with dyslexia who escaped the cut‐off criteria of being severe dyslexic increased a little suggests that the intervention contributed to the normalization of the reading and spelling performance of some of the children.

The second research question was how phonological awareness, rapid automatized naming, letter knowledge, and verbal working memory predicted children's responsiveness to the sustained intervention and to what extent reading and spelling outcomes of the sustained intervention can be explained from initial reading and spelling abilities at the start of the intervention and initial treatment success after the declarative phase of the intervention. There is consensus that reading efficiency is preceded by reading accuracy (Barth et al., 2009; Jenkins, Fuchs, van den Broek, Espin, & Deno, 2003; Torgesen et al., 2001) and that word reading accuracy plays an important role in the establishment of reading fluency (Barth et al., 2009; Torgesen et al., 2001). This would imply that incomplete skills of decoding accuracy are related to a weak decoding efficiency achievement and that some predictors of reading accuracy are related to reading efficiency as well. The findings of the present study were partly in line with the assumption. Although a study by Tijms (2011) revealed that the effectiveness of a Dutch intervention was to a large extent robust to individual differences in precursor measures, the present study showed the role of some precursor measures in relation to children's responsiveness to intervention. For word and pseudoword decoding efficiency, for example, rapid automatized naming ability at the start of the intervention directly predicted posttest, an expected finding, because reading fluency is often related to proficiency on rapid automatized naming tasks (Furnes & Samuelsson, 2011; Scheltinga et al., 2010; Zoccolotti et al., 2014). Additionally, reading efficiency outcomes were predicted by children's initial abilities at the start of the intervention directly and indirectly via Time 2, and directly by their initial treatment success on efficiency tasks. Such autoregressive effects on word reading efficiency replicates findings by Scheltinga et al. (2010). We demonstrated some overlap in individual differences in predicting posttest outcomes in reading efficiency and in reading accuracy. As we found for efficiency, we have also demonstrated that children's initial abilities at pretest predicted posttest directly on word and pseudoword decoding accuracy. In line with a study by Otaiba and Fuchs (2002), suggesting that the degree of responsiveness to intervention was characterized by poor phonological awareness, we found a direct effect of phoneme deletion (the ability to manipulate sounds in spoken words) to the posttest of pseudoword decoding accuracy. Although other studies usually describe intervention effects in terms of reading or spelling, the present study included both reading and spelling analyses. Phoneme deletion predicted the posttest of spelling, and in line with other studies, the prediction of rapid automatized naming on posttest spelling was not significant. In an active production, as in word spelling, the appeal on a child's fast serial naming seems restricted and thus related to word spelling in a limited way (Furnes & Samuelsson, 2011). Regarding individual variation, the results of the present study are in line with a study by Frijters et al. (2011) who also evidenced that phonological awareness and rapid automatized naming are important predictors of responsiveness to intervention in reading. With regard to the second research question, we can conclude that children's responsiveness to the sustained intervention implies an important role of phonological awareness in reading accuracy and rapid automatized naming in the prediction of reading efficiency. Additionally, their reading outcomes can to a large part be explained from children's initial reading abilities at the start of the intervention. The role of their initial treatment success during the first phase of the intervention, in relation to posttest outcomes, is limited to word and pseudoword decoding efficiency and spelling.

There are some limitations to the present study. Ideally, we had used a control group in which children without dyslexia, without the intervention, were included to function as a second control group. In future research, it would be interesting to find out the role of natural growth of children and to compare results of children with dyslexia in our study with an appropriate control group who did not receive intervention. Ethical principles and the Dutch dyslexia approach made it very difficult to create such a control group in the present study. Although this group was not available, we made an attempt to interpret the results of the present study in terms of effects by using *z*‐scores and cut‐off scores. It should also be acknowledged that the present study was an attempt to connect precursor measures to the responsiveness to intervention in reading and spelling and also to connect children's initial reading and spelling abilities, and their initial treatment success to reading and spelling outcomes at posttest. In addition to other studies that revealed the relation of precursor measures to intervention outcomes, this study contributes to existing knowledge, in understanding individual differences in reading and spelling outcomes after a systematic, specialized, and sustained intervention in children with dyslexia. When further studying these relations, it is recommended to also incorporate a second control group. Another important question that still remains is about the explicit focus on learning to visualize the GPCs into symbolic scaffolds in the first phase of the intervention. The assumption that symbolic scaffolds simplify the recognition of the structure of words and are important in reading and spelling accuracy and efficiency is not demonstrated yet. The positive results may suggest that children benefit from the simplification, but to be able to demonstrate this assumption, children's change in skills would need to be compared with a group of children who did not learn the symbolic scaffolds during a sustained dyslexia intervention. It can be questioned to what extent the explicit focus on learning the GPCs, the symbolic scaffolds, and on the phonological segmentation has speeded children up. One could also argue that this decoding approach has slowed them down to some extent, as continuous decoding strategies take more time to read and spell words. Therefore, for future research, it is recommended to investigate the role of the use of symbolic scaffolds and syllabic segmentation in more depth. Finally, it should be kept in mind that the possibility to generalize the results of the present study to other languages may be difficult. Orthographies differ in depth, whereas Dutch is considered as a transparent orthography (Frost, 2012; Landerl et al., 2012; Landerl & Wimmer, 2008).

In sum, we can conclude that children with dyslexia benefited from a two‐phase sustained reading and spelling intervention. The degree of children's responsiveness to the sustained intervention in reading and spelling accuracy could be predicted from their phonological awareness at the start of the intervention. Their responsiveness in reading efficiency was predicted by the rapid automatized naming. Reading and spelling outcomes of the sustained intervention could additionally directly and/or indirectly be predicted by children's reading abilities at the start of the intervention and their initial treatment success.
